# A Proposed Dental Reform

**Published:** 1883-06

**Authors:** C. M. Wright

**Affiliations:** Cincinnati


*Communications—Continued from Page 272.*

### A Proposed Dental Reform.

[A Legal Safeguard in the Dance of Death.]

BY PROF. C. M. WRIGHT, CINCINNATI.

In the worlds of Politics, Law, Morals and Medicine, there is always the demand for reforms. We have read for years of Civil Service Reform, of Municipal Reform, of Judiciary Reform. Thousands of earnest men and women are advocating different reforms in dress, in morals, in education. The Medical Profession is always contriving sanitary reform for the benefit of the people, and reforms for its own conduct and practices. Dentistry has not been behind Law, Religion, or Medicine in *suggestions* for reforms. In all these departments, however, there are a thousand demands, prayers and plans, where a *single real step* is taken toward the goal. Hope dies with youth, generally, and incredulity and conservatism follow. We get used to talking about, and listening to talk, about reform. We learn by experience that it is only *talk*, and that the best laid plans are forgotten, and succeeded by others as soon as the breath gets cold, that but a few hours before had been warmed by an apparently everlasting good purpose. Proposed reforms are too often like the rocket, rushing heavenward in a blaze of glory—coming down like the sparks, without having effected anything, or like the stick, which is utterly lost and forgotten. Here and there, once in a thousand or so times, a reform is proposed, accepted, and *accomplished.*

The Mad River Valley Dental Society believe that such a thing was born at its meeting in Dayton on the 22d of May. Ohio inaugurated successful Dental Legislation some years ago. Neighboring States sneered at her for a time, but fifteen of them have followed her example. Ohio now proposes to make an effort to "protect the public" from the danger of taking "Laughing Gas," "Vitalized Air," "Ether" and other anæsthetics from any reckless and ignorant fellow who may have *muscle* enough to "draw a tooth." If a man puts "Dentist" on his sign, and

"Teeth Extracted without Pain," the people believe that he is qualified, and that the dental profession back him in his practice. They consequently go to his office, and with lamb-like confidence allow him to carry them to the brink of the grave and hold them over it while they are insensible. If they come back they are quite happy, cry and laugh hysterically awhile, and relate dreams as a rescued drowning man does. It is to the credit of the people that they do not often wake up in the surly mood of Rogue Riderhood and demand the satisfaction of the law. In most cases they are willing to write down their names in a big book, signifying in common with hundreds of others, that they too have escaped death at the hands of the dentist. Now, the profession know that people *have* gone straight from the dental chair to mansions in the skies. The profession know that many hundreds of people have "never felt well" since they took that little trip with the dentist to where the gates were ajar. The profession know that the man who plays Othello every day, and suffocates the dear people to within an inch of their lives "without the slightest fear"—does this because he is so blissfully ignorant about the physiological effects of the gas or ether on the heart, or brain, or blood, or lungs of his snoring patient. The profession know, that in a majority of cases, this confident operator could not tell by any sort of an examination whether his patient had any disease that should warn him to special caution or not—could not do anything to help the patient out of the grave if he should happen to slip in while being held over it. The profession know that at the coroner's inquest, liable to be held after the administration of the harmless (?) laughing gas, the reckless dentist could only testify: "The man stopped breathing and didn't begin again—that's all." "In ten minutes I had a physician in, but the man was dead." The profession know that unless this doctor had happened to have given *special* study to these cases, neither he nor the dentist would do what *might* bring back this traveler "from that bourne, etc." It is impossible for one poor little human mind to know everything about the complex subject of disease, and the greatest student of medicine, the best trained general practitioner of medicine might mistake a case of *Hœmor-*

*rhagic Variola* for *Purpura Hœmorrhagica*, while an experienced Pest House doctor could recognize it at a glance. This fact should be utilized when practical, and the dental profession believe that it is practical to require *special* study and *special* qualification in the man who dares to take his patients on a "dance of death" for the extraction of teeth. A committee was appointed by the society that met at Dayton, to bring this matter before other dental societies in the State, and to look out that the stick shall not come down till the rocket has illuminated the subject for the benefit of the people and the State legislators. They propose to urge the enactment of a law, compelling every dentist who wishes to administer anæsthetics, to appear before the State Board of Dental Examiners, now in office by a previous law, and to satisfy this Board that he is qualified by proper special study and experience, to be trusted with the lives of our citizens. The people would rise up and demand this safeguard if they were familiar with the literature and all the circumstances of the case as it now stands. The Dental Profession owes this to itself, for it has been criminally careless. It should not wait till the horse has been stolen from our own locality, before locking the stable door. When a man or woman dies in a dental chair, every dentist will have blood on his hands, "which, like the stains on the hands of the guilty Macbeth, all ocean's waters will never wash out."

